# 

**Published:** 1877-01

**Authors:** 


**Subscription Blank Enclosed.**

Vol. XII. JAN., 1877. No. 4

### THE BISTOURY:

SURG GENLS OFFS

### A QUARTERLY JOURNAL,

*Devoted to the Exposition of Charlatanism in Medicine and the Education of the People upon Medical Subjects!*

### Thad S. Up de Graff, M. D., Editor.

*Terms of Subscription, Fifty-five Cents per Annum, in Advance*

ELMIRA, N. Y.

PRINTED FOR THE PROPRIETOR, AT THE DAILY ADVERTISER JOB PRINTING HOUSE.

**READ THE ADVERTISEMENTS.**

